# Entomopathogenic fungus treatment changes the gut bacterial diversity of *Rhipicephalus microplus* ticks

**DOI:** 10.1186/s13071-023-05790-5

**Published:** 2023-06-06

**Authors:** Emily Mesquita, Diogo Paes da Costa, Laura Nóbrega Meirelles, Mariana Guedes Camargo, Thaís Almeida Corrêa, Vânia Rita Elias Pinheiro Bittencourt, Irene da Silva Coelho, Huarrisson Azevedo Santos, Richard Alan Humber, Patrícia Silva Golo

**Affiliations:** 1 Postgraduate Program in Veterinary Sciences, Veterinary Institute, Federal Rural University of Rio de Janeiro, Seropédica, Brazil; 2 Microbiology and Enzymology Laboratory, Federal University of Agreste Pernambuco, Garanhuns, PE 55292-270 Brazil; 3 Department of Animal Parasitology, Veterinary Institute, Federal Rural University of Rio de Janeiro, Seropédica, RJ Brazil; 4 Department of Veterinary Microbiology and Immunology, Veterinary Institute, Federal Rural University of Rio de Janeiro, Seropédica, RJ Brazil; 5 Department of Epidemiology and Public Health, Veterinary Institute, Federal Rural University of Rio de Janeiro, Seropédica, RJ Brazil; 6 USDA-ARS Emerging Pests and Pathogens Research, R. W. Holley Center for Agriculture and Health, Ithaca, NY 14850 USA

**Keywords:** Vector-borne diseases, Biological control, Bovine diseases, *Metarhizium anisopliae*, 16S rRNA, Tetracycline

## Abstract

**Background:**

Ticks are obligate bloodsucking parasites responsible for significant economic losses and concerns with human and animal health, mainly due to the transmission of pathogens. Entomopathogenic fungi have been intensively studied as an alternative strategy for tick control that can be used in combination with synthetic acaricides in the integrated management of ticks. Here, we investigated how the gut bacterial community of *Rhipicephalus microplus* is shaped after *Metarhizium anisopliae* treatment and how the tick susceptibility to the fungus is affected after disrupting gut bacterial microbiota.

**Methods:**

Partially engorged tick females were artificially fed with pure bovine blood or blood plus tetracycline. Two other groups received the same diet and were topically treated with *M. anisopliae*. The guts were dissected, and the genomic DNA was extracted 3 days after the treatment; the V3–V4 variable region of the bacterial 16S rRNA gene was amplified.

**Results:**

The gut of ticks that received no antibiotic but were treated with *M. anisopliae* exhibited lower bacterial diversity and a higher occurrence of *Coxiella* species. The Simpson diversity index and Pielou equability coefficient were higher in the gut bacterial community when *R. microplus* were fed with tetracycline and fungus-treated. Ticks from fungus-treated groups (with or without tetracycline) exhibited lower survival than untreated females. Previous feeding of ticks with the antibiotic did not change their susceptibility to the fungus. *Ehrlichia* spp. were not detected in the gueated groups.

**Conclusions:**

These findings suggest that myco-acaricidal action would not be impacted if the calf hosting these ticks is under antibiotic therapy. Moreover, the hypothesis that entomopathogenic fungi can affect the bacterial community in the gut of *R. microplus* engorged females is endorsed by the fact that ticks exposed to *M. anisopliae* exhibited a dramatic reduction in bacterial diversity. This is the first report of an entomopathogenic fungus affecting the tick gut microbiota.

**Graphical Abstract:**

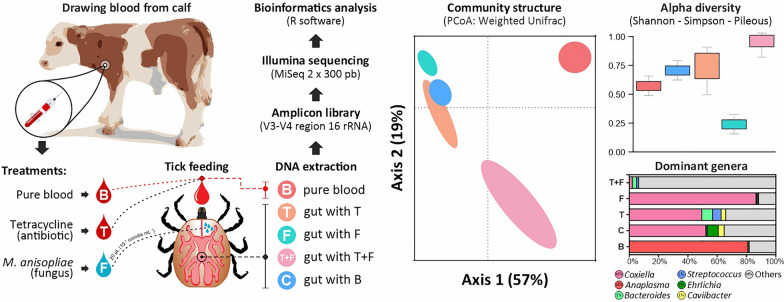

**Supplementary Information:**

The online version contains supplementary material available at 10.1186/s13071-023-05790-5.

## Background

The importance of the microbiome of ticks with medical and veterinary significance has been increasingly recognized in recent years [1–3]. Most of these studies are driven by the importance of understanding tick-borne diseases to improve their control. As bloodsucking obligate ectoparasites, ticks must rely on endosymbionts for nutritional supplementation [4–6]. *Rhipicephalus microplus*, considered the most widely distributed tick in tropical areas [7], is a one-host tick species that preferentially parasitizes cattle, causing great economic losses in livestock mainly due to the transmission of hemoparasites such as *Babesia bovis*, *Babesia bigemina*, and *Anaplasma marginale* [8, 9].

Antibiotic treatments have been used either on vertebrate hosts or directly into ticks through artificial feeding or injection to understand the role of the gut microbiome in the biology of ticks and tick-borne diseases [10–12]. It was shown that the composition of the gut microbiota in a tick could affect the acquisition, colonization, and transmission of tick-borne pathogens [13]. When the gut microbiota was altered in *Ixodes scapularis*, the colonization of *Borrelia burgdorferi* was reduced [11]. Nevertheless, the opposite is also suggested to happen. Adegoke et al. [14] demonstrated that when *R. microplus* was infected with an apicomplexan, *Theileria* sp., the gut microbiome was altered, and its diversity, species richness, and evenness were lower than in uninfected ticks.

Synthetic acaricide applications are usually the method of choice for tick control but have raised concerns about human, animal, and environmental health and have led to the emergence of resistant tick populations [15]. The use of entomopathogenic fungi is a promising alternative when seeking a safer and more sustainable method for tick control [16]. The entomopathogenic fungal genus *Metarhizium* includes several species that are among the most explored and successfully utilized biopesticides in agriculture [17], with the potential to be used against ticks commercially. Fungal spores infect ticks upon contact and can be used in integrated pest management, reducing synthetic acaricides overuse, according to previous studies [16, 18, 19]. Despite this, much is left to be examined to fully understand tick–fungi interactions, especially regarding the immune and biochemical responses of fungus-infected ticks [20–25]. As far as we know, there is no report connecting tick gut bacteria and the action of fungal entomopathogens. Could the tick microbiota influence its susceptibility to entomopathogenic fungi? In insects, the answer seems to be host-dependent: For the mosquito *Anopheles stephensi* and the beetle *Dendroctonus valens*, the host’s microbiota positively contributed to fungal action [26, 27], which was not demonstrated for the German cockroach *Blattella germanica* [28].

For instance, what is known about tick microbiome interactions is mainly related to its physiology and the impacts on the biology of tick-borne diseases. The first description of *R. microplus* bacterial diversity was in 2011 by Andreotti et al. [29], and more recently, authors have been describing more findings according to different geographic locations [30]. In the present study, we aimed to explore how the initial steps of the entomopathogenic fungal infection, after topical treatment, may trigger gut bacterial community changes in *R. microplus* and whether gut bacterial community disruption impacts the susceptibility of that tick to entomopathogenic fungi.

## Methods

### Artificial feeding of *Rhipicephalus microplus* females

One calf was artificially infested with *R. microplus* larvae (Porto Alegre strain) (Reck et al. [15] and held at the W.O. Neitz Parasitological Research Station at the Federal Rural University of Rio de Janeiro (UFRRJ), Brazil (CEUA [Ethics Committee on the Use of Animals]/Veterinary Institute, UFRRJ—protocol no. 9714220419). Ticks were naturally fed on the calf for 19 or 20 days and then manually removed, carefully detaching them from the host skin (to avoid disrupting the mouth parts). The calf used was not under antibiotic or acaricidal therapy for 2 months before the experiment. Artificial feeding of *R. microplus* was adapted from Valim et al. [31] and Ribeiro et al. [32]. Partially engorged tick females were weighed, surface sterilized with sodium hypochlorite (0.05% v/v) for 3 min and dried with paper towels. The blood used to artificially feed the ticks was directly collected from the jugular vein of the same calf (CEUA/Veterinary Institute, UFRRJ—protocol no. 6407270619) where ticks were naturally fed through a vacuum system into a 3.6 ml tube containing citrate as an anticoagulant (Vacuplast, Turkey). Tick females weighing approximately 30–70 mg were artificially fed with pure blood or blood plus tetracycline hydrochloride (Merck, Darmstadt, DE) at 0.05 mg ml^−1^ for 7 h using plastic tips at 37 ± 1 °C and ≥ 80% relative humidity (RH). Tips were individually filled with blood (up to 50 µl) every hour, as much as necessary. Partially engorged females were allowed to feed with an average of 350 µl of blood at most. Ticks were individually weighed before and after artificial feeding to measure blood uptake. Only ticks that had doubled their initial weight were considered for further analysis (0.03 µg of tetracycline mg^−1^ female weight) [12].

### *Metarhizium anisopliae* fungal suspension

The fungal isolate *Metarhizium anisopliae* sensu stricto LCM S04 [19] was used in the present study. The cultures were cultivated on oat medium under controlled conditions (25 ± 1 °C; ≥ 80% RH) for 21 days. Conidia were suspended in a solution of sterile distilled water with polyoxyethylene sorbitan monooleate (Tween^®^ 80) (Isofar, Rio de Janeiro, Brazil) 0.01% (v/v) at 1 × 10^8^ conidia ml^−1^. Fungal viability was assessed by plating an aliquot of 20 µl of 1 × 10^5^ conidia ml^−1^ of the same fungal suspension on potato dextrose agar (PDA) (Kasvi, Paraná, Brazil). Conidial germination was determined 24 h after incubation at 25 ± 1 °C and RH ≥ 80% using an optical microscope (×400) (ECLIPSE E200; Nikon, Tokyo, Japan). A minimum of 300 conidia were evaluated, and the percent germination was calculated. Conidia were considered germinated when the germ tube was visible. The fungal suspensions used in the experiments had viability of at least 95%. As the present study accessed Brazilian genetic heritage, the research was registered at the National System for the Management of Genetic Heritage and Associated Traditional Knowledge (SisGen) under the code AA47CB6.

### Assays for the control of *Rhipicephalus microplus* under antibiotic therapy

Ticks artificially fed with pure blood or blood plus tetracycline (“Artificial feeding of *Rhipicephalus microplus* females” section) were topically treated with *M. anisopliae* suspension. Four groups of 10 females each were established as follows: untreated ticks fed with pure blood (control group) (ctrl); untreated ticks fed with blood plus tetracycline (T); fungus-treated ticks previously fed with pure blood (F); fungus-treated ticks previously fed with blood plus tetracycline (T+F). As soon as the artificial feeding was finished, ticks were washed in tap water to remove any residual blood, dried, and weighed. Then, ticks that had doubled their weight were tape-fixed in Petri dishes using doubled-sided tape and topically treated with 20 µl of 1 × 10^8^ conidia ml^−1^. The suspension was applied on the tick’s dorsal region, and ticks were kept at 25 ± 1 °C and RH ≥ 80%. Seventy-two hours after the fungus treatment, the guts of three females of each group were dissected for DNA extraction. Survival of the other ticks was recorded daily for 15 days. This bioassay was performed three times with new batches of conidia and *R. microplus* ticks.

### *Rhipicephalus microplus* dissection and gut DNA extraction

The guts of *R. microplus* females were dissected with sterile tweezers and scalpel blades using sterile phosphate-buffered saline solution (PBS) [130 mM NaCl, 1 mM KH_2_PO_4_, 5.6 mM Na_2_HPO_4_, 2 mM KCl (pH 7.2)]. Removed gut tissues were washed twice in sterile PBS and kept in RNA later (Thermo Fisher Scientific, Waltham, MA, USA) at −80 °C until extraction. First, tick guts were frozen in liquid nitrogen and macerated with a sterile pestle. The DNA of the gut homogenate was extracted following the protocol of the DNeasy Blood & Tissue kit according to the manufacturer’s instructions (QIAGEN Inc., Valencia, CA, USA). The DNA of the blood from the calf (B) used for the natural and artificial feeding was also extracted according to the same protocol mentioned above.

### Library preparation and 16S rRNA sequencing

The V3–V4 variable region of the bacterial 16S rRNA gene was amplified for genomic DNA from 13 samples (triplicates from gut samples of each group and one for blood control [B]), using the primers Bakt_341F (CCTACGGGNGGCWGCAG) and Bakt_805R (GACTACHVGGGTATCTAATCC) [33]. The Herculase II Fusion DNA polymerase (Agilent Technologies, Inc., Santa Clara, CA, USA) and the Nextera XT Index Kit v2 (Illumina, Inc., San Diego, CA, USA) (300-base-pair [bp] paired-end reads) were used on the Illumina^®^ MiSeq^®^ platform with a 30% PhiX spike on Macrogen (Seoul, South Korea). The binary base calls were converted in FASTQ format, sequences were demultiplexed, and barcodes were removed using the bcl2fastq v2.20 package (Illumina Inc., San Diego, CA, USA).

### Sequence analysis

Adapters were removed from the raw data (1,250,293 forward and reverse sequences), which were then filtered based on quality scores and trimmed using the DADA2 Pipeline version 1.16 [34] in R version 4.1.1 (R Core Team 2022) in conjunction with RStudio 1.4.1717 (RStudio Team 2022) [35]. The FIGARO tool [36] was used to calculate the optimized truncation parameters. Forward and reverse reads were truncated at 270 bp and 215 bp, respectively. Forward and reverse reads with more than two expected errors were discarded, respectively, and reads were truncated at the first instance of a quality score ≤ 2. The read error rates were learned by the “learnErrors” function, alternating between error rate estimation and sample inference until convergence. The amplicon sequence variants (ASVs) were inferred using the “given” function, and sequences were merged by the “mergePairs” function. The chimeras were removed from collections of unique sequences by the method of consensus across samples using the “removeBimeraDenovo” function. T taxonomic assignments were given based on the SILVA SSU 132-modified database [37] using the “IdTaxa” function from the DECIPHER v 2.20 R package [38], a method with classification performance that is better than the standard naïve Bayesian classifier method [39]. Sequences assigned to mitochondrial genome, chloroplasts, and non-bacteria were removed. After these procedures, 3313 ASVs were assigned to the remaining 839,263 bacterial sequences for the rarefaction procedure and statistical analysis.

### Statistical and bioinformatics analysis

The tick survival curve was analyzed by a log-rank test with a significance level of 0.05 using GraphPad Prism version 8.4.2 (GraphPad Software, San Diego, CA, USA). All other statistical analyses were performed in R software version 4.1.1 (R Development Core Team, Vienna, Austria) in conjunction with RStudio 1.4.1717 (Posit Software, Boston, MA).

Multivariate exploratory analyses were done using the “vegan” R package version 2.5-7 [40]. Beta diversity was studied based on principal coordinate analysis (PCoA) using the weighted UniFrac distance matrix of the microbial communities in each sample, showing differences between bacterial communities from different treatments. The predominance of rare, specialist, and generalist ASVs was assessed by the multinomial species classification method (CLAM) with adjustment for multiple comparisons, using the supermajority specialization threshold (*K* = 2/3, *P* = 0.05) [41]. The graphs were constructed with the ggplot2 R package version 3.3.3 [42].

The analysis of the network between ASVs was evaluated using bootstrap estimates of SparCC correlation by SpiecEasi R package version 1.1.0, resulting in node and edge matrices [43]. Only edges with significant correlations (*P* < 0.01) were selected for graphical construction using Gephi software version 0.9.2 [44], highlighting the number of connections (degree), betweenness centrality (BC), and the sign of the correlations.

## Results

### Tetracycline antibiotic therapy did not affect tick survival

**Fig. 1:**
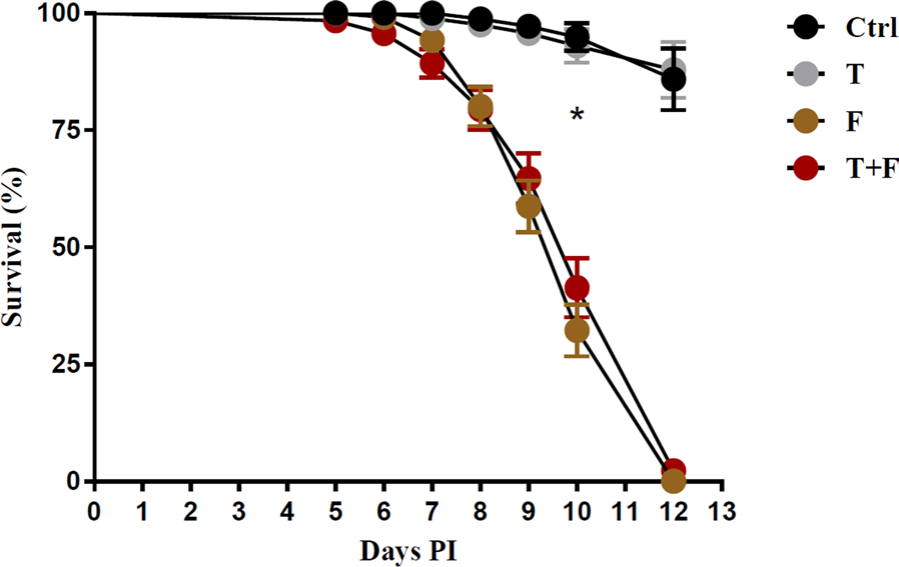
Survival of *Rhipicephalus microplus* females after artificial blood-feeding with or without tetracycline and *Metarhizium anisopliae* treatment (average and standard error). Asterisk indicates a statistical difference between ctrl and T+F (*P* < 0.05) by long-rank test. Treatments: ctrl—fungus-untreated ticks previously fed with pure blood (control group); T—fungus-untreated ticks previously fed with blood plus tetracycline; F—fungus-treated ticks previously fed with pure blood; T+F—fungus-treated ticks previously fed with blood plus tetracycline

Tick survival in the ctrl group was higher than that in the F (*χ*^2^ = 81.9, *P* < 0.0001) and T+F (*χ*^2^ = 68.4, *P* < 0.0001) groups, but was not different from the T group (*χ*^2^ = 0.06, *P* = 0.80). F and T+F survival were similar (*χ*^2^ = 0.5, *P* = 0.47) (Fig. 1). Ticks from both fungus-treated groups (with and without tetracycline) were dead (0% survival) within 12 days. At the same time, the fungus-untreated groups (ctrl and T) exhibited an average of 85% survival. No difference in survival was observed between ticks artificially fed with tetracycline (T) or not (ctrl).

### Bacterial community structure and diversity

**Fig. 2:**
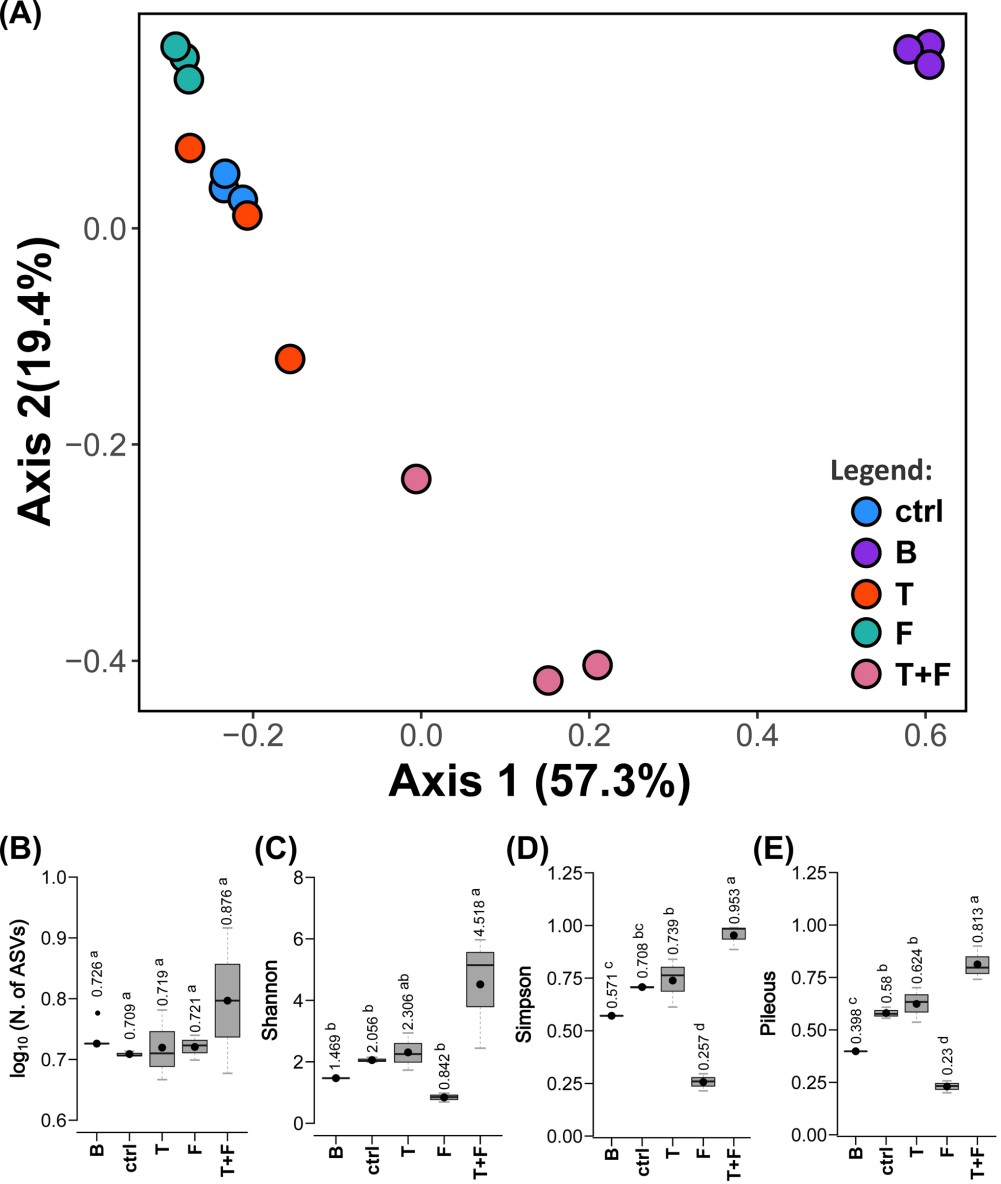
Structure and alpha diversity of bacterial communities in *Rhipicephalus microplus* guts and calf’s blood. **A** PCoA-based community beta diversity analysis, based on the UniFrac distance weighted matrix for ASVs, showing the differences between the groups. **B** Count of unique ASVs. **C** Shannon's diversity index. **D** Simpson's diversity index. **E** Pielou equability coefficient. The dots indicate the exact location of the means. **B**–**E** Treatments with means followed by the same superscript lowercase letters do not differ from each other by the Tukey honestly significant difference (HSD) test at a 5% significance level. Group designations are given in Fig. 1

PCoA, based on the weighted UniFrac distance matrix, explained about 77% of the total variance of the multivariate model across the two principal axes only (Fig. 2A). By this parameter, the gut bacterial community of ticks fed with tetracycline and treated with *M. anisopliae* (T+F) differed from the other niches. The gut of ticks fed with blood plus tetracycline without fungal treatment (T) or fed with pure blood and treated with fungus (F) exhibited bacterial community structures relatively close to each other. The communities of the former group (i.e., T) were also close to that observed in the gut of ticks from the ctrl group (ticks fed with pure blood). The bacterial community of the calf’s blood (B) was the most distinct.

Treatments exhibited similar average numbers of bacterial ASVs in the analyses of the beta diversity (Fig. 2B). In agreement with the PCoA result, Shannon's diversity index (Fig. 2C), Simpson's diversity (Fig. 2D), and Pielou equability coefficient (Fig. 2E) [45, 46] measured in the gut of T+F were the highest and differed significantly from those observed in the other treatments, except for Shannon's index, where T was not different from T+F. Overall, F exhibited the lowest indices of gut bacterial diversity, followed by B, ctrl, T, until peaking at T+F.

### Bacterial community composition

**Fig. 3:**
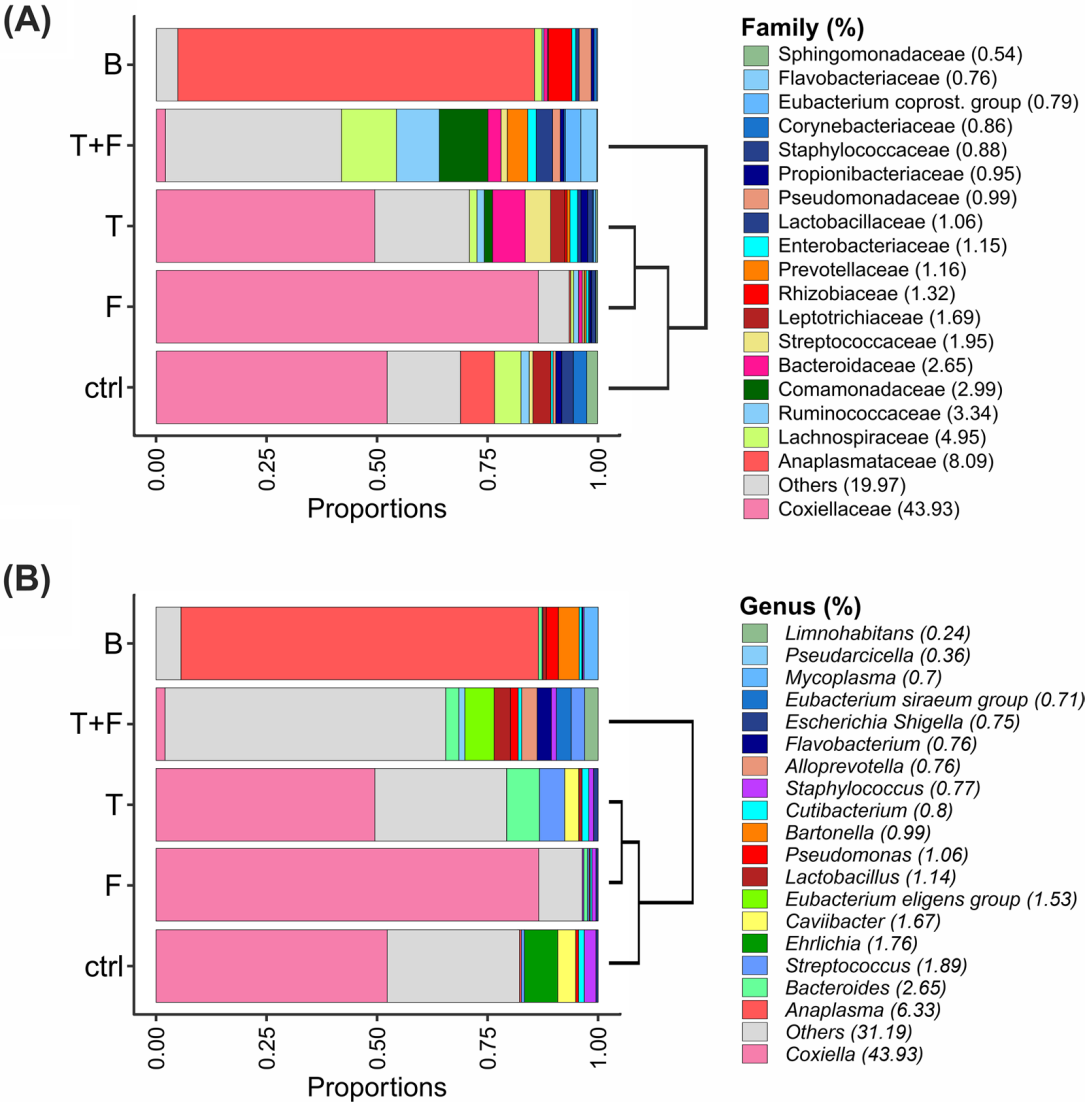
Composition of predominant **A** bacterial families and **B** genera in the ticks’ guts and pure blood (B). The samples were grouped as dendrograms according to the distance calculated using the Spearman correlation coefficient. Group designations are given in Fig. 1

The taxonomic profiling generated 839,263 quality-filtered bacterial sequences classified into 3313 ASVs. The ASV classification coverage in the taxonomic ranks was as follows: phylum (96%), class (94%), order (86%), family (75%), genus (49%), and species (5%). The 19 most abundant families accounted for more than 80% of the total families (Fig. 3A). In all treatments, the most abundant families (above 2% of total ASVs) were Coxiellaceae (43.9% of the sequences), followed by Anaplasmataceae (8.1%), Lachnospiraceae (4.9%), Ruminococcaceae (3.3%), Comamonadaceae (3.0%), and Bacteroidaceae (2.6%).

Following the families, the 19 most abundant genera accounted for more than 80% of the total genera (Fig. 3B). The most abundant genera (more than 1% of total ASVs) were *Coxiella* (43.9%), *Anaplasma* (6.3%), *Bacteroides* (2.6%), *Streptococcus* (1.9%), *Ehrlichia* (1.8%), *Caviibacter* (1.7%), *Eubacterium* (1.5%), *Lactobacillus* (1.1%), and *Pseudomonas* (1.1%). Except for ASVs assigned to *Anaplasma* and *Ehrlichia*, both from the family *Anaplasmataceae*, all these genera were representatives of different families.

According to Spearman's correlation coefficient, supporting PCoA results (Fig. 2A), the compositions of bacterial families (Fig. 3A) and genera (Fig. 3B) in B and in the ticks’ gut from T+F differed from those observed in the other treatments, especially ctrl. While in the other treatments (i.e., ctrl, T, and F), bacteria of *Coxiellaceae*, mostly referable to *Coxiella*, were predominant, T+F guts exhibited a predominance of species from *Lachnospiraceae*, followed by species of *Comamonadaceae* and *Ruminococcaceae*. In addition, data from the guts of T+F treatment had a cluster of several other bacterial families with frequencies of occurrence lower than 0.5%. In B, *Anaplasmataceae* (mostly in the genus *Anaplasma*) was predominant, followed by *Bartonellaceae* (primarily assigned to *Bartonella*).

### Niche occupancy

**Fig. 4:**
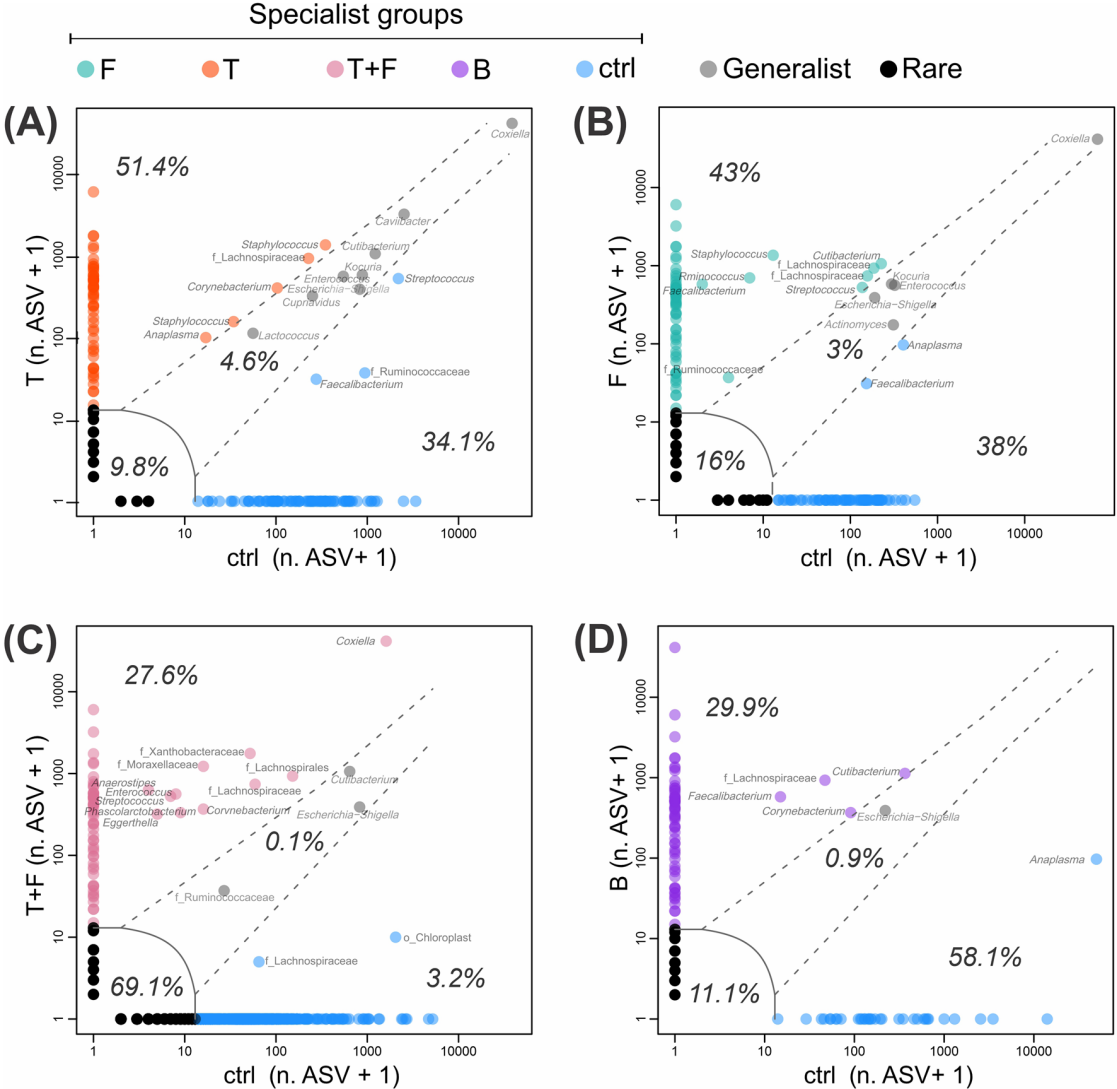
Multinomial species classification method (CLAM) for the niche occupancy test. Bacterial genera are shown only in circles that stand out significantly in each habitat. The generalists (gray), specialists (orange, blue, green, pink, and purple), and rare (black) are indicated with their respective percentages. Percentage values represent the direct count of unique ASVs in each niche. **A** T vs. ctrl; **B** F vs. ctrl; **C** T+F vs. ctrl; **D** blood vs. ctrl. Group designations are given in Fig. 1

According to the multinomial species classification method (CLAM), enrichment up to the bacterial class level was observed (Additional file 1: Fig. S1). In general, bacteria from the class *Gammaproteobacteria* were predominant in all treatments. According to the CLAM, contrasts with the ctrl indicated enrichment of *Bacilli* and *Clostridia* in F (Additional file 1: Fig. S1A), *Clostridia* and *Actinobacteria* classes in T+F (Additional file 1: Fig. S1B), and *Alphaproteobacteria* and *Bacilli* in B (Additional file 1: Fig. S1C). Enrichment up to the generic level was also observed, contrasting the niche occupancy of each treatment with the ctrl (Fig. 4). Depending on the comparison, the enriched groups (specialist bacteria) in the ctrl group varied, with a predominance of *Faecalibacterium*, *Anaplasma*, and *Streptococcus* (Fig. 4).

The guts of group T exhibited significant enrichment of specialized bacteria (51.4%), highlighting ASVs associated with *Staphylococcus*, *Corynebacterium*, *Anaplasma*, and other species of *Lachnospiraceae* (Fig. 4A). In the guts of fungus-treated females (F), the specialist bacteria accounted for 43% of the total ASVs, with *Cutibacterium*, *Streptococcus*, *Staphylococcus*, *Ruminococcus*, *Faecalibacterium*, and other ASVs from the *Lachnospiraceae* and *Ruminococcaceae* families standing out (Fig. 4B). The guts from T+F exhibited specialist bacterial species were limited to only 27.6%, highlighting the enrichment of the genera *Enterococcus*, *Streptococcus*, *Corynebacterium*, *Anaerostipes*, *Phascolarctobacterium*, *Eggerthella*, and others associated with the families *Lachnospiraceae*, *Xanthobacteraceae*, and *Moraxellaceae* (Fig. 4C). The bacterial communities of calf’s blood (B) and the guts of ticks fed exclusively on blood (ctrl) were also compared, which indicated a predominance of specialists at rates of 29.9% and 58.1%, respectively (Fig. 4D). In this case, significant enrichment of ASVs associated with the genera *Cutibacterium*, *Faecalibacterium*, and *Corynebacterium* was observed in the blood compared with the ctrl.

### Co-occurrence network analysis

**Fig. 5:**
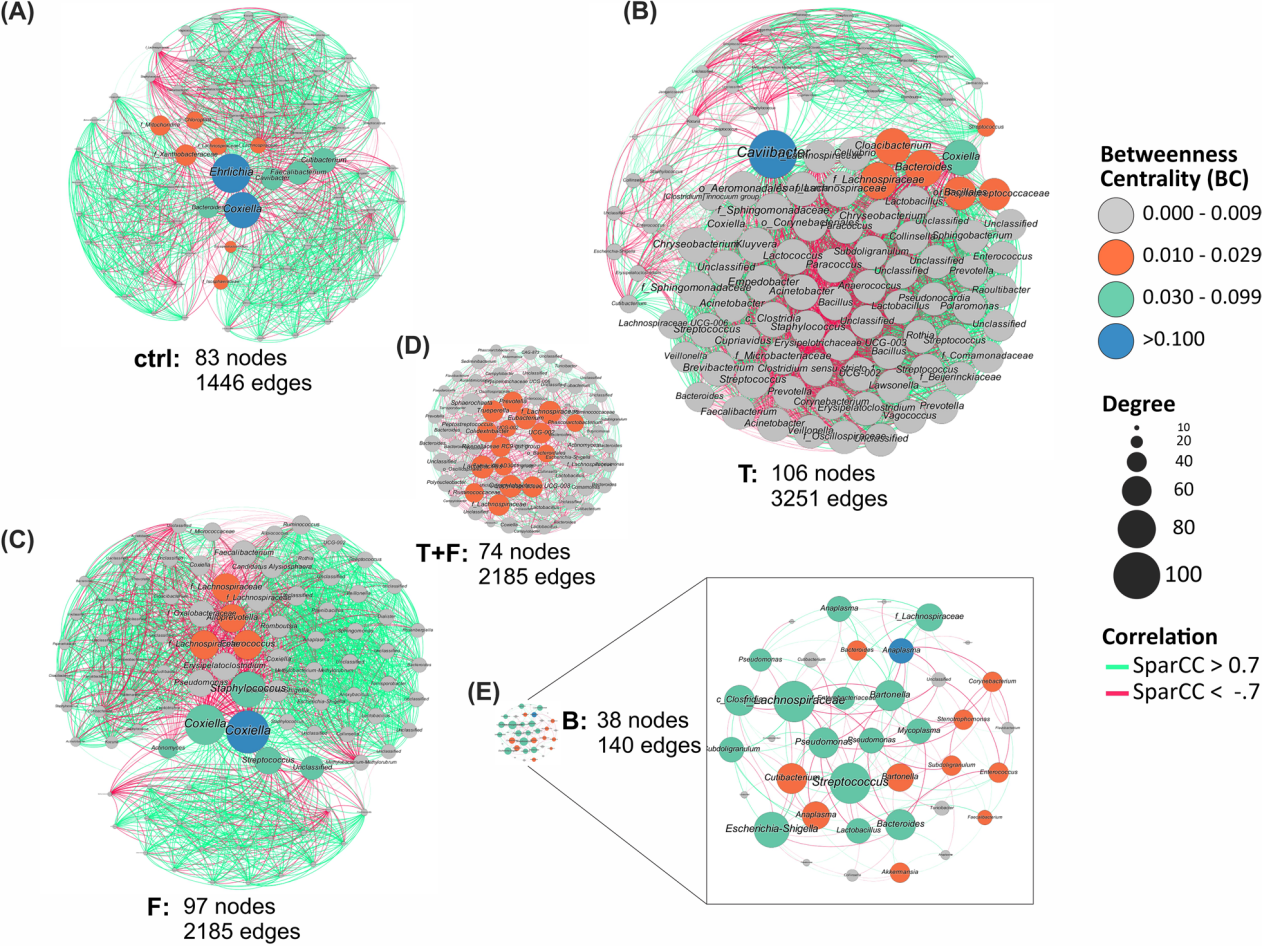
Network co-occurrence analysis of the bacterial communities in *Rhipicephalus microplus* guts treated or not with *Metarhizium anisopliae* based on the 16S rRNA gene. The size of the nodes is proportional to the degree, and the colors indicate discrete intervals of betweenness centrality (BC). Group designations are given in Fig. 1

Co-occurrence analysis of ASVs revealed key species (keystone species) that maintained bacterial communities in the different samples studied (Fig. 5, Table 1). The gut bacterial communities of untreated ticks (ctrl) were mainly connected by two keystone species from the genera *Ehrlichia* and *Coxiella*. Four ASVs associated with the genera *Cutibacterium*, *Faecalibacterium*, *Caviibacter*, and *Bacteroides* also played a role (Fig. 5A). This network had 83 nodes, 1446 edges, and 68.19% of positive interactions (Table 1).

**Table 1:** Network metrics of the bacterial communities in the gut of ticks and in the pure blood of calves

Parameters	ctrl	T	F	T+F	B
Number of nodes^a^	83	106	97	74	38
Number of edges^b^	1446	3251	2185	1247	140
Positive edges^c^	68.19	54.57	67.19	44.03	48.57
Negative edges^d^	31.81	45.43	32.81	55.97	51.43
Connected components^e^	1	1	1	1	1
Network diameter^f^	2	3	2	2	4
Graph density^g^	0.425	0.584	0.469	0.462	0.199
Modularity^h^	0.450	0.192	0.377	0.225	0.248
Average degree^i^	34.843	61.34	45.052	33.703	7.368
Avg. clustering coefficient^j^	0.875	0.866	0.875	0.590	0.340
Avg. path length^k^	1.575	1.422	1.531	1.538	2.104
Maximum degree^l^	81	104	93	44	15

^a^Number of unique ASVs present on the network

^b^No. of significant correlations between ASVs (weight ≥ 0.7 and *P*-value < 0.05)

^c^Percentage of positive correlations

^d^Percentage of negative correlations

^e^No. of separate components in the network

^f^Shortest distance between the two most distant nodes in the network

^g^Measure of how many ties between ASVs exist compared with how many ties between ASVs are possible

^h^Measures the strength of division of a network into clusters or communities

^i^Average no. of edges per node

^j^Measures the weighted degree to which nodes in a graph tend to cluster together

^k^Average no. of steps along the shortest paths for all possible pairs of nodes

^l^Maximum number of connections (edges) observed

Treatments: B—pure blood sample from the calf; ctrl—ticks artificially fed with pure blood; T—ticks artificially fed with blood plus Tetracycline; F—ticks artificially fed with pure blood and treated with *Metarhizium anisopliae*; T+F—combined use of tetracycline and *M. anisopliae* treatment

The bacterial community from T had the highest network complexity (nodes = 106, edges = 3251, positive correlations = 54.57%). Keystone species were associated with an ASV of *Caviibacter*, followed by *Coxiella*, and to a lesser extent with *Cloacibacterium*, *Bacteroides*, and other species from the *Lachnospiraceae* and *Peptostreptococcaceae* families (Fig. 5B, Table 1).

The network from F showed the second-highest complexity (nodes = 97, edges = 2185, positive correlations = 67.19%), highlighting as keystone species two ASVs from the genus *Coxiella*, followed by *Staphylococcus*, *Streptococcus*, and *Actinomyces* (Fig. 5C, Table 1). In ticks that were fed with tetracycline and treated with fungus (T+F), the network complexity and the number of positive correlations were lower (nodes = 74, edges = 1247, positive correlations = 44.03%) compared to those observed in the single treatments (ctrl, T, or F) (Fig. 5D). In the case of ticks fed with tetracycline and treated with fungus (T+F), several ASVs had similar centralities and degrees of connection. This highlights the participation of many genera, including *Lactobacillus*, *Eubacterium*, *Colidextribacter*, *Prevotella*, *Trueperella*, *Campylobacter*, and *Phascolarctobacterium*.

As expected, the bacterial community network of the calf’s blood (B) was the least complex (nodes = 38, edges = 140, positive correlations = 48.57%). This network was regulated by several ASVs, mainly those associated with *Streptococcus*, *Pseudomonas*, *Escherichia*/*Shigella*, *Bacteroides*, *Bartonella*, *Lactobacillus*, *Anaplasma*, and *Mycoplasma*, as well as one ASV associated with the Lachnospiraceae family (Fig. 5E).

## Discussion

Antibiotics play a crucial role in treating infectious diseases such as clinical mastitis [47] and tick-borne diseases [48], and they can be used as a growth promoter [49]. Nevertheless, the impact of antibiotic administration on the susceptibility of ticks to fungal treatment is yet to be elucidated. In the present study, ticks were artificially fed with tetracycline and topically treated with an entomopathogenic fungus. Interestingly, similar survival was observed in the fungus-treated females whether they were previously fed with the antibiotic or not. According to the Merck Sharp & Dohme Corp. (MSD) veterinary manual, treatment with oxytetracycline for cattle is 10 mg^−1^ kg^−1^ day^−1^. Here, the tetracycline concentration added to the bovine blood for the artificial tick feeding followed the 30 mg^−1^ kg^−1^ day^−1^ proportion, based on a previous study [12]. Accordingly, even when allowed to feed on a blood meal with a higher antibiotic concentration, the susceptibility of ticks to *M. anisopliae* was not affected. This suggested that when cattle are under antibiotic therapy with tetracycline, the susceptibility of female ticks would not be affected.

Bacterial communities in ticks treated with antibiotic and fungus (T+F) had the highest proportion of sequences that were too rare to classify, i.e., the number of sequences representing these bacterial species was not sufficient to be considered in the analysis. Therefore, it is possible that these treatments together allowed the enrichment of a wide array of bacterial species. In addition, the niche occupancy analysis showed a higher number of specialist species than generalists for all treated groups in comparison to the ctrl, suggesting that all treatments could disturb the bacterial community at different levels. Analysis of the relative participation of bacterial class (CLAM) (Fig. 4) revealed that the bacterial taxa in F were also observed in T+F; however, the sequence reads of the five most abundant taxa were higher in F than in T+F. These facts thus explain the different diversity indexes between these groups. Additionally, there was an increase in the number of taxa in T+F that changed its bacterial composition in comparison to F. Discrepancies between F and T+F in network co-occurrence analysis (Fig. 5) indicate how the interactions between bacteria and keystone species in each group varied. The T+F group exhibited the highest diversity index (Fig. 3) in contrast with the lowest number of keystone species and bacterial interactions. Keystone species are not necessarily abundant, but they have a strong impact on other species based on the number of interactions [50, 51]. Accordingly, keystone species can shape the bacterial community composition because of their strong connections. Despite T+F data exhibiting a higher number of species (i.e., increased diversity index), these species did not establish solid interactions, probably because they are opportunistic bacteria that only arose due to disruption caused by the fungus and antibiotic treatments together.

Endosymbiotic microorganisms have an important role in obligate hematophagous arthropods, providing nutrients that are scarce in a blood diet [4, 52]. Duron et al. [5] reported that *Ornithodoros moubata* is dependent on the endosymbiont *Francisella*, responsible for vitamin B synthesis. Guizzo et al. [12] demonstrated that for *R. microplus*, *Coxiella* is an endosymbiont critical for metanymph maturation and tick physiology. These authors also showed that *Coxiella* was abundant in different tissues of *R. microplus* females, with predominance in the ovary and Malpighian tubules but very low levels in the gut. On the other hand, in our study, *Coxiella* was the most abundant bacterial genus in *R. microplus* guts in all groups except T+F. The higher abundance of this taxon in F suggested that the fungal infection allows *Coxiella* enrichment in the tick gut, decreasing other taxa and reducing bacterial diversity. This result was also observed with *Anopheles stephensi* after *Beauveria bassiana* treatment [27]. These authors reported that the symbiont *Serratia marcescens* increased in the mosquito gut after the fungus treatment. In contrast, here, guts from T+F exhibited the lowest *Coxiella* abundance and the highest diversity. In this group, the reduced presence of *Coxiella* probably resulted from the combination of tetracycline administration (a broad-spectrum agent that inhibits bacterial protein synthesis) [53] plus the fungal treatment. *Coxiella* reductions appear to allow the incidence of other bacteria in the gut, which could explain the increased diversity. In addition, some bacteria reported in the tick gut bacterial community have been suggested to be resistant to tetracycline [54]. This fact might explain why *Coxiella* abundance was not reduced in tetracycline-fed ticks (T) when compared with ctrl, since protein associated with resistance has been found in *Coxiella burnetii* [55].

As far as we know, this is the first report of tick–gut bacteria interactions with an entomopathogenic fungus. Previous studies [26, 56] with different insect species reported that changes in the gut microbiota could improve or impair the entomopathogenic fungal action depending on the arthropod host. This outcome could be due to variations in the microbiota composition of different hosts. Here, tick survival of females treated with the fungus that did or did not receive the antibiotic (F or T+F) was similar. Analogous results were observed by Ramirez et al. (2018) [57] with *Aedes aegypti*. According to these authors, the reduced gut bacterial load did not change the entomopathogenic fungal virulence, even when using a high fungal inoculum load. It is possible that these cleared bacteria (bacteria removed after the antibiotic treatment) did not influence the success or failure of the fungal infection. However, this study focused on testing only adult females of *R. microplus*, and therefore the effects of bacterial community disruption in immature phases might have a different outcome.

The present study analyzed the guts of ticks 3 days after *M. anisopliae* treatment. This time was chosen based on previously unpublished work [Mesquita, E.; Golo, P.S.] demonstrating that, after 72 h, LCM S04 conidia would have already germinated and penetrated the *R. microplus* cuticle, reaching the tick’s internal organs. Additionally, fungal actions cause deterioration in the arthropod body over time that hampers dissection methods. Accordingly, what might have happened after 72 h regarding bacterial–fungal interactions remains to be elucidated. *Coxiella*, *Ehrlichia*, *Caviibacter*, *Cutibacterium*, and *Escherichia*/*Shigella* were the most common genera observed in the guts of the ctrl group. The blood sample and the ctrl group shared only 0.9% of the sequences, followed by ctrl and T+F, with 0.1% of generalists. Bacteria identified in the guts can be inherited mainly through transovarial transmission, across generations, and through host skin [4]. Although the calf used here demonstrated no clinical signs of anaplasmosis, molecular analysis of the blood sample showed that *Anaplasma* was the most abundant bacterial genus found in that animal’s blood (Fig. 3). However, this fact only indicates that the abundance of *Anaplasma* in the blood was higher than that of the other bacteria, and not necessarily a high level of infection. This loss of taxa between the host’s blood and the tick may occur through the blood digestion process, since the tick gut has defense strategies against invasive microorganisms. This defense is mainly driven by hemoglobin antimicrobial-derived fragments called hemocidins [58]. Besides this, gut defense mechanisms include molecules such as antimicrobial peptides and possibly reactive oxygen species [59].

The genus *Ehrlichia* was found in the guts from the ctrl group but not detected in F, T, or T+F. In 2016, Cabezas-Cruz et al. [60] described a new species, *Ehrlichia minasensis*, isolated from *R. microplus* hemolymph, pathogenic for cattle. To date, *E. minasensis* and *E. ruminantium* are the only species known to infect cattle naturally [61, 62]. Even though the identification of the *Ehrlichia* species or possible implications in its life cycle were not addressed in the present study, the non-detection of this genus in the gut of fungus-treated ticks supports the hypothesis that entomopathogenic fungal infection can negatively impact the occurrence of a bacterium that causes tick-borne disease. Studies have reported effects of entomopathogenic fungi in the transmission and life cycle of vector-borne pathogens after treating the arthropod with the fungus (viz., *Glossina fuscipes fuscipes* and *Trypanosoma congolense*) [63] as well as of *Anopheles gambiae* and *Plasmodium falciparum* [64]. Nevertheless, to our knowledge, any literature regarding tick–pathogen–fungus interactions, especially compared to studies with insects, is nonexistent. Here, the disruption of the tick gut microbiota by the entomopathogenic fungus *Metarhizium* (combined or not with the antibiotic) indicates an exploitable feature of this fungus in its use against ticks. However, the analysis here was centered only in the tick gut, and no other tick tissues. Accordingly, further investigation is warranted on the tripartite interactions between ticks versus pathogens versus fungi.

## Conclusions

Challenging *R. microplus* with *M. anisopliae* changes the tick gut bacterial community mainly by increasing the enrichment of the endosymbiont *Coxiella.* Tetracycline administration plus *M. anisopliae* treatment leads to a dramatic reduction in the population of *Coxiella* and alters the *R. microplus* gut bacterial community by increasing its bacterial diversity. Nevertheless, antibiotic therapy does not influence tick susceptibility to the entomopathogenic fungus.

## Supplementary Information

**Additional file 1: Fig. S1.** Relative participation of bacterial class in each composer niche on the network according to the multinomial species classification method (CLAM). Percentage values within the boxes were calculated over a raw ASV scale for each niche. Comparisons of the T (**A**), T+F (**B**), and B (**C**) treatments with the control were highlighted because of the greater contrasts. The percentages based on the log (ASV+1) transformation are on the vertical axis in parentheses. Treatments: ctrl—fungus-untreated ticks previously fed with pure blood (control group); F—fungus-treated ticks previously fed with pure blood; T+F—fungus-treated ticks previously fed with blood plus tetracycline; B—pure blood sample from the calf.

## Data Availability

The paired sequences and BioSamples assignment from this study are available in the NCBI repository under the project code PRJNA849240.
