# Supplementary figures and images for: Entomopathogenic fungus treatment changes the gut bacterial diversity of Rhipicephalus microplus ticks
Source: Parasit Vectors. 2023 Jun 6;16:185. doi: 10.1186/s13071-023-05790-5 (PMC10245507; doi:10.1186/s13071-023-05790-5)

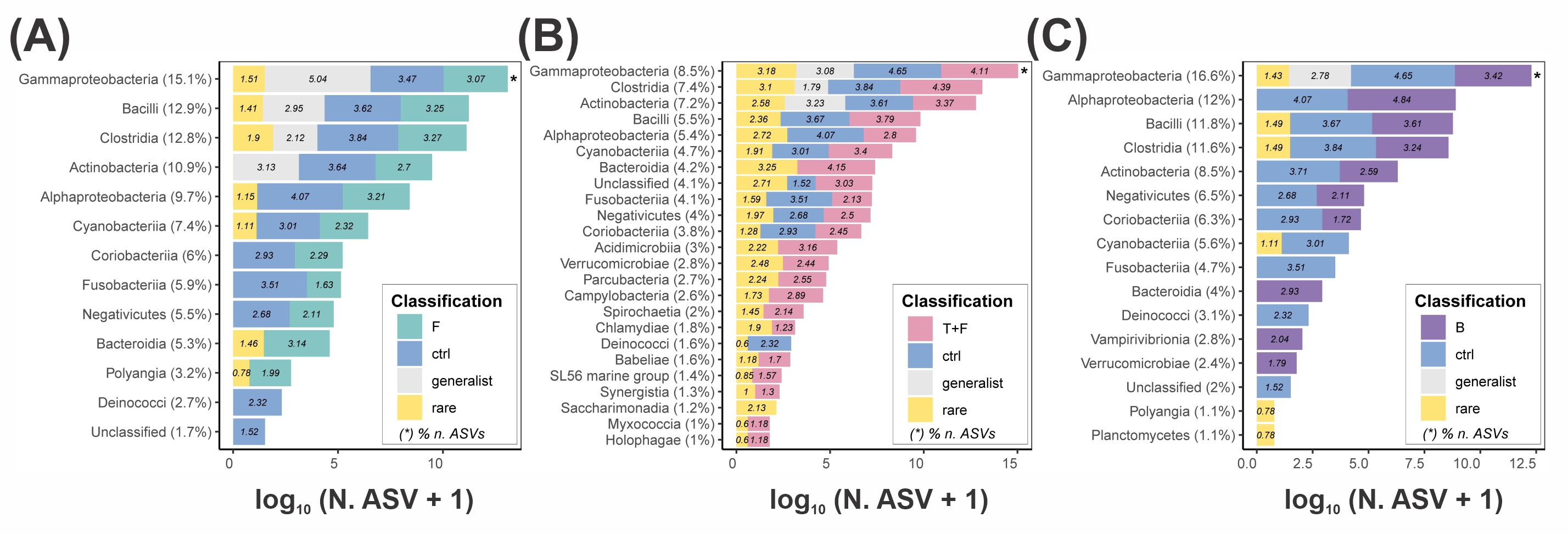

Supplement: Supplementary file 1 — Additional file 1: Fig. S1. Relative participation of bacterial class in each composer niche on the network according to the multinomial species classification method (CLAM). Percentage values within the boxes were calculated over a raw ASV scale for each niche. Comparisons of the T (A), T+F (B), and B (C) treatments with the control were highlighted because of the greater contrasts. The percentages based on the log (ASV+1) transformation are on the vertical axis in parentheses. Treatments: ctrl—fungus-untreated ticks previously fed with pure blood (control group); F—fungus-treated ticks previously fed with pure blood; T+F—fungus-treated ticks previously fed with blood plus tetracycline; B—pure blood sample from the calf. [file 13071_2023_5790_MOESM1_ESM.jpg]
